# Supplementary material for: The interactome of intact mitochondria by cross-linking mass spectrometry provides evidence for coexisting respiratory supercomplexes
Source: Mol Cell Proteomics. 2017 Dec 8;17(2):216–32. doi: 10.1074/mcp.RA117.000470 (PMC5795388; doi:10.1074/mcp.RA117.000470)
Supplement: Supplemental Data [file supp_RA117.000470_133922_0_supp_23978_fzffwf.zip › spectra_annotation/mito_spectra_annotation/410-1-16-1-15-1.pdf]

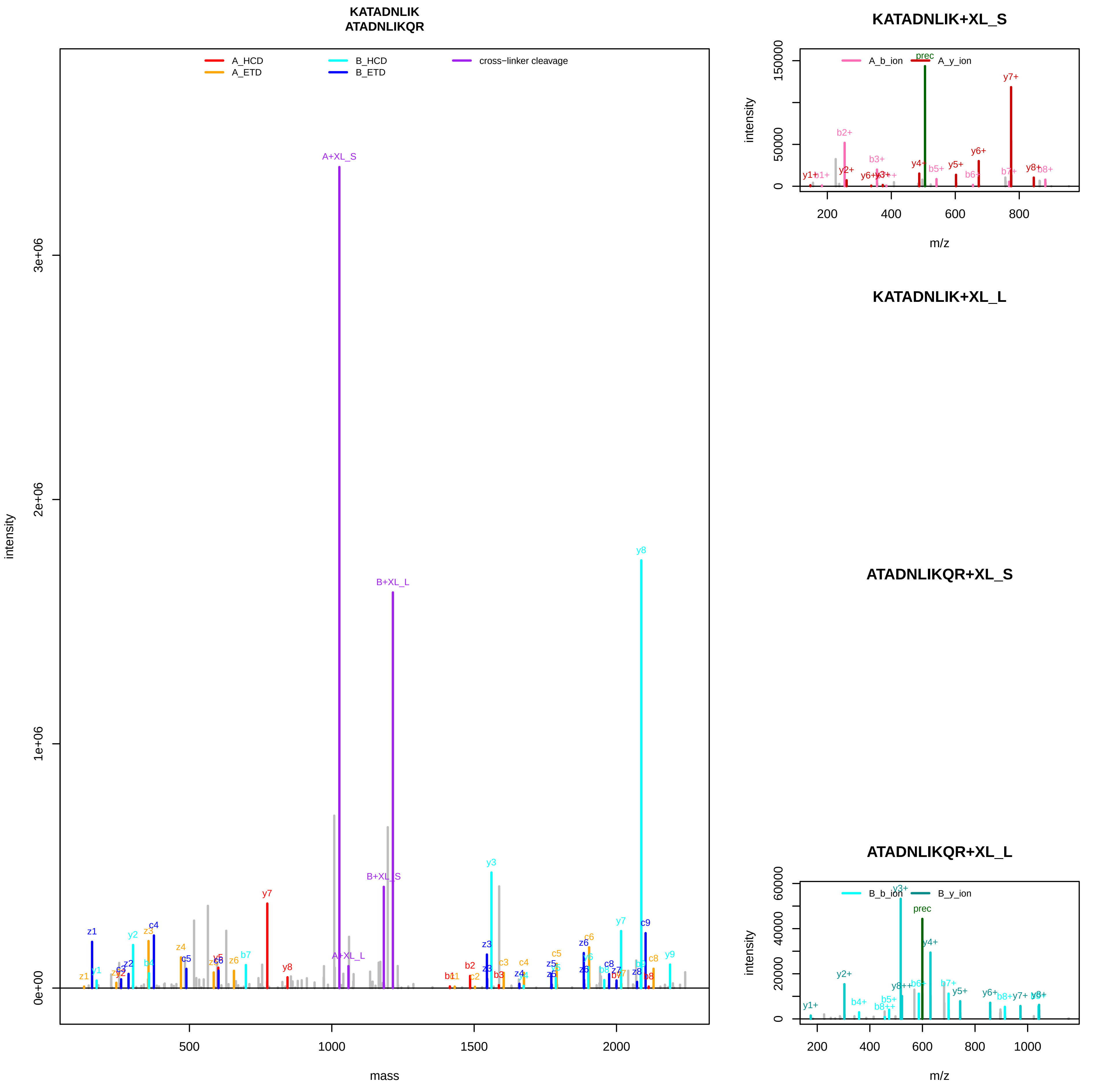
KATADNLIK
ATADNLIKQR
A_HCD
A_ETD
B_HCD
B_ETD
cross−linker cleavage
A+XL_S
B+XL_L
B+XL_S
A+XL_L
intensity
mass
0e+00
1e+06
2e+06
3e+06
500
1000
1500
2000
y8
y3
y7
y9
c9
z6
c6
z1
y2
z3
c4
KATADNLIK+XL_S
A_b_ion
A_y_ion
prec
y7+
b2+
b3+
y6+
y4+
y5+
y2+
y8+
b8+
b7+
b6+
b5+
y1+
b1+
m/z
0
50000
150000
200
400
600
800
KATADNLIK+XL_L
ATADNLIKQR+XL_S
ATADNLIKQR+XL_L
B_b_ion
B_y_ion
y3+
y4+
y2+
b4+
b5+
b6+
b7+
y5+
y6+
y7+
y8+
y1+
0
20000
40000
60000
1000
